# Archaeometric classification of ancient human fossil bones, with particular attention to their carbonate content, using chemometrics, thermogravimetry and ICP emission

**DOI:** 10.1186/1752-153X-8-26

**Published:** 2014-04-25

**Authors:** Mauro Tomassetti, Federico Marini, Luigi Campanella, Alfredo Coppa

**Affiliations:** 1 Department of Chemistry, University of Rome “La Sapienza”, P.le Aldo Moro, 5-00185 Rome, Italy; 2 Department of Environmental Biology, University of Rome “La Sapienza”, P.le Aldo Moro, 5-00185 Rome, Italy

**Keywords:** Thermal analysis, Fossil bones, Inductively coupled plasma (ICP), Principal component analysis (PCA), Carbonate thermal decomposition

## Abstract

**Background:**

The potential of coupling chemometric data processing techniques to thermal analysis for formulating an “archaeometric” classification of fossil bones was investigated. Moreover, the possibility of integrating the outcomes of this approach with the results of inductively coupled plasma (ICP) emission spectroscopy for an anthropological interpretation of the observed patterns was also examined.

**Results:**

Several fossil bone samples coming from the necropolis of El Geili, in the middle Nile, an important archaeological site, were first of all subjected to thermogravimetric (TG) and derivative thermogravimetric (DTG) analysis and the main steps of the curves were analyzed. This allowed fossil bone samples to be differentiated, both by means of classical bidimensional and chemometric representations, namely Principal Component Analysis (PCA). In particular, two clusters were observed, attributable to samples of different antiquity. In addition, inductively coupled plasma (ICP) emission spectroscopy showed that the samples in the cluster corresponding to more recent burials are characterized by a higher Zn content, suggesting a more varied diet.

**Conclusions:**

The experimental data obtained using thermogravimetry (TG-DTG) allows us to differentiate all the fossil bone samples analyzed into two separate clusters and to interpret this differentiation in terms of the observed transitions.

## Background

Thermogravimetric curves (TG-DTG) of several human fossil bone samples, all belonging to the necropolis of El Geili [1] have been recorded. In this archeological site, burials from different ages (at least covering the period 3000 B.C.-400 A.D., according to the literature [1]) have been unearthed. An in-depth analysis of the mass losses was carried out as a function of temperature during a linear scan in the range 25–1000°C, focusing in particular on the decomposition processes of carbonate materials [2,3]. Indeed, not only has been the carbonate content in the samples determined, but also it was possible to differentiate between traces of the carbonate originally present in the hydroxyapatite matrix and the so-called “re-formation” or “secondary” carbonate, i.e., the carbonate of pedogenic origin which is formed by reprecipitation on the buried bones as a result of soil processes [4]. In this respect, to characterize the analyze samples we propose a chemometric representation base on Principal Component Analysis which is compared to the ones based only on the relative amount of the two types of carbonate or on carbonate and collagen. The results obtained were then examined with the aim of formulating an “archaeometric” classification of the analyzed samples on the basis of the considerations reported in the literature in this regard [2-6]. The main TG/DTG [7,8] outcomes were then organized into a data matrix that was processed by exploratory chemometric [9] methods, which allowed a satisfactory classification of all the samples into two homogeneous clusters. A quantitative analysis of the calcium, zinc and strontium content of these samples determined using ICP spectroscopy was also carried out. Strontium *vs* zinc and calcium *vs* strontium contents [10-12] (which can be plotted as binary diagrams) provided a further confirmation of the archaeometric evaluation based on thermal analysis but, above all, allowed important anthropologic considerations to be formulated regarding the food habits and the sociological status of the individuals the fossil finds belonged to.

## Results and Discussion

Several old bone samples, taken from the El Geili necropolis, which is known to contain tombs of various periods, both prehistoric and belonging to the early centuries of the Christian age [1], were subjected to TG and DTG analysis.

**Figure 1:**
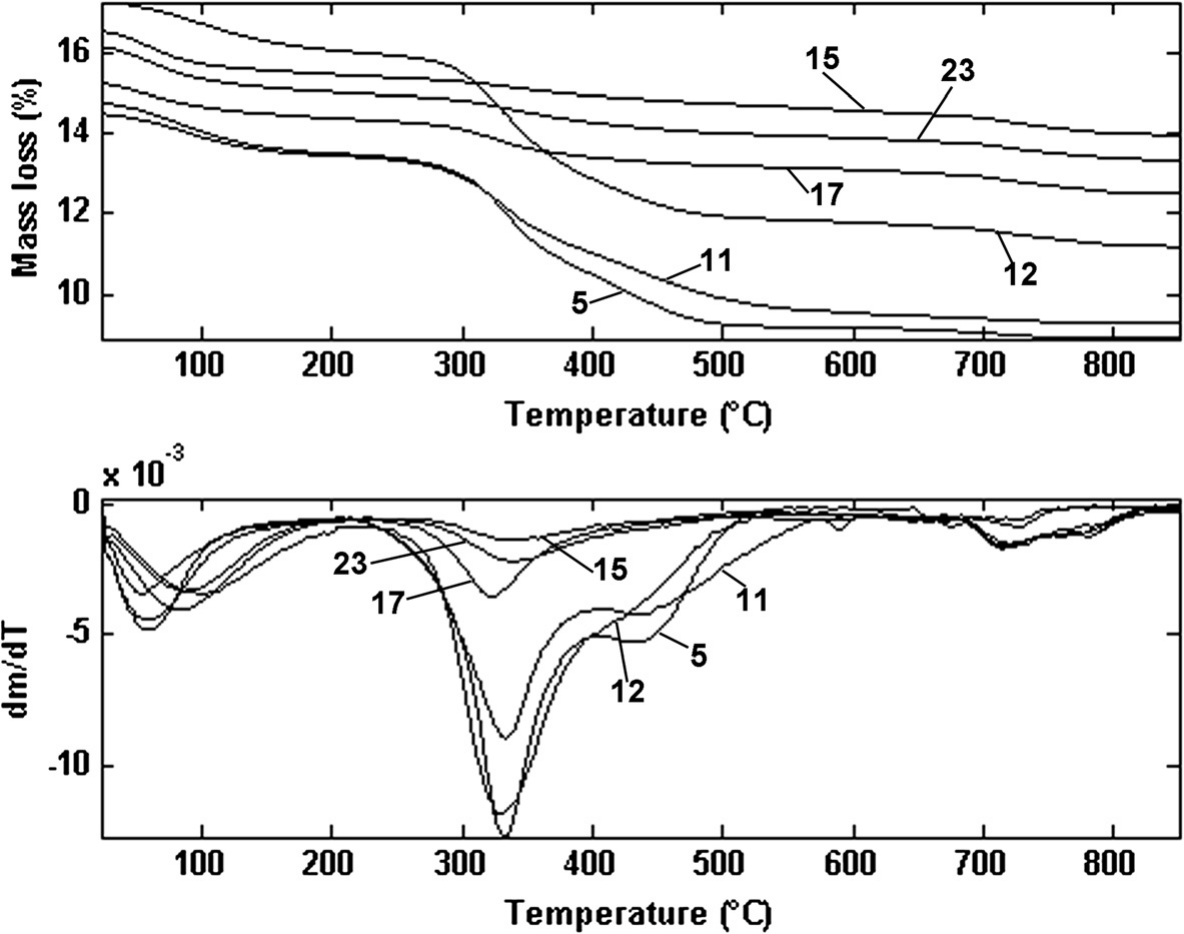
**TG and DTG profiles of typical bone samples showing collagen and carbonates decomposition steps; heating rate 10°C** **min**^**-1 **^**under an air stream of 100 cm**^**3**^ **min**^**-1 **^**(The numbers in Figure refer to sample indices).**

Typical examples of recorded TG-DTG curves are shown in Figure 1. The main steps in the thermogravimetric curves were linked: (a) to moisture loss, (b) to organic components (i.e. collagen) decomposition, sometimes including two partly overlapped DTG peaks, at about 330 and 460°C, respectively, and sometimes only one DTG peak at about 335°C.

At higher temperatures, different TG-DTG steps are evidenced between about 600 and 850°C, all linked to carbonate decomposition from carbonated hydroxylapatite [4].

The main thermal data (i.e. mass loss and temperatures, recorded in the principal steps of collagen and carbonate thermal decomposition) are set out in Table 1.

**Table 1:** Summary of the main thermal analytical data obtained by TG-DTG

**Sample number**	**Step (a)**	**Step (b)**		**Step (c)**		**% TG residue at 1000°C**
		**Between about 190°C and 550°C**		**Between about 600°C and 850°C**		
		**(Collagen decomposition process)**		**Substep c(1)**	**Substep c(2)**	
				**Between about 600°C and 750°C**	**Between about 750°C and 850°C**	
				**(secondary carbonate decomposition)**	**(original carbonate decomposition)**	
	**% H**_**2**_**O loss**	**Main DTG peak T°C**	**Total % mass loss**	**% mass loss**	**% mass loss**	
1	7.6	333	29.3	1.5	0.4	61.1
2	8.3	332	28.0	1.7	0.3	61.3
3	6.7	335	28.5	1.4	0.3	62.7
4	7.1	329	30.6	1.6	0.4	59.1
5	8.6	334	29.1	1.5		60.0
6	8.2	330	27.0	1.1		62.5
7	6.1	330	31.4	1.4		61.1
8	8.2	331	26.9	1.5	0.4	62.4
9	8.3	334	28.2	1.4		61.6
10	8.1	335	28.6	1.6	0.4	60.6
11	7.3	335	26.3	2.0		63.8
12	7.1	335	24.1	2.6	1.0	64.7
13	6.9	333	27.7	2.1		62.5
14	4.8	340	5.4		1.5	87.4
15	6.6	330	4.3	3.1	0.8	83.8
16	7.6	330	8.0	3.5	1.2	79.2
17	5.6	325	7.8	3.5	1.0	81.4
18	6.8	340	5.1	3.0	0.8	83.7
19	6.0	345	3.1	3.3	0.8	86.0
20	5.9	350	4.0	2.8		86.1
21	5.9	330	6.1	2.7	0.4	83.4
22	6.6	325	5.9	3.6	1.5	82.0
23	7.2	350	6.1	2.7	0.9	82.4

RSD% ≤ 1.2 for all T values.

RSD% ≤ 1.5 for all % mass loss values and residue at 1000°C.

**Figure 2:**
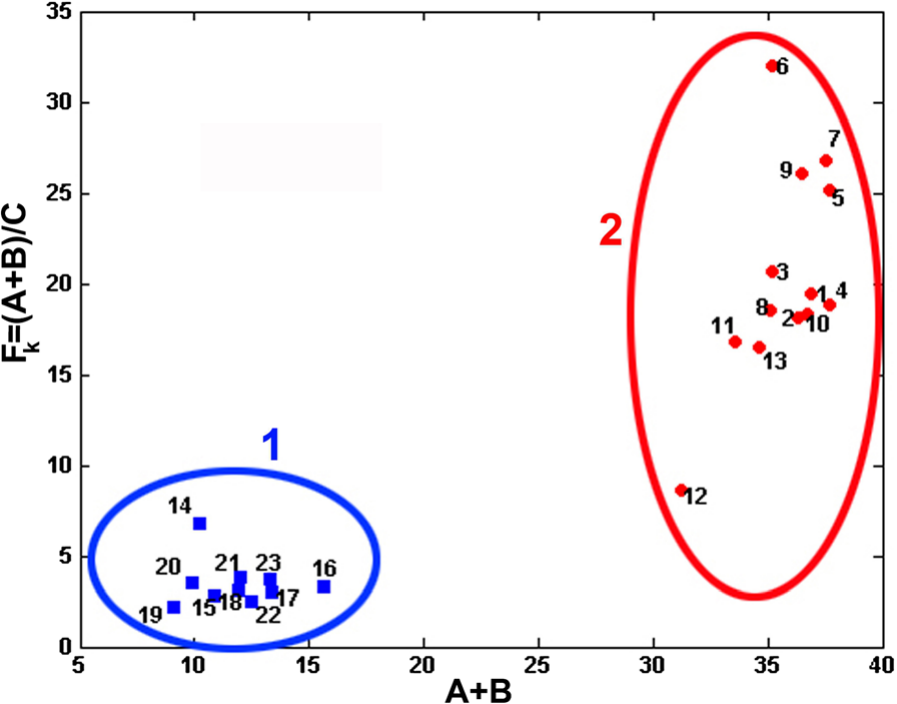
**Graphical representation of thermal data according to the two parameters proposed by Szoor [**[13]**].**

G. Szoor [13] actually carried out an interesting investigation in which it was attempted, with some success, to identify thermogravimetric parameters that could be correlated with the antiquity of the sample. His principal observation was essentially that, with increasing bone sample age, the percentage of carbonate contained in it increased while the percentage of collagen decreased. Accordingly he introduced two parameters: a coefficient (A + B) calculated on the basis of the mass loss referring to the sum of the two first thermogravimetric processes (related respectively, (A) to moisture loss and (B) to all collagen decomposition) and a fossilization coefficient (denoted as F_k_), essentially obtained from the quotient between (A + B) and (C), where (C) is the mass loss linked to all carbonate decomposition. These two parameters (see Table 2) were computed using data from Table 1 and represent two parameters suggested by Szoor and represented in a binary diagram (Figure 2).

**Table 2:** Szoor Parameters: original and simplified as proposed in this study

**Sample number**	**Szoor’s steps**			**Szoor’s parameters**		**Simplified parameters**	
	**A**	**B**	**C**	**A + B**	**(A + B)/C**	**B**	**B/C**
1	7.6	29.3	1.9	36.9	19.4	29.3	15.4
2	8.3	28.0	2.0	36.3	18.1	28.0	14.0
3	6.7	28.5	1.7	35.2	20.7	28.5	16.8
4	7.1	30.6	2.0	37.7	18.9	30.6	15.3
5	8.6	29.1	1.5	37.7	25.1	29.1	19.4
6	8.2	27.0	1.1	35.2	32.0	27.0	24.5
7	6.1	31.4	1.4	37.5	26.8	31.4	22.4
8	8.2	26.9	1.9	35.1	18.5	26.9	14.2
9	8.3	28.2	1.4	36.5	26.1	28.2	20.1
10	8.1	28.6	2.0	36.7	18.3	28.6	14.3
11	7.3	26.3	2.0	33.6	16.8	26.3	13.1
12	7.1	24.1	3.6	31.2	8.67	24.1	6.69
13	6.9	27.7	2.1	34.6	16.5	27.7	13.2
14	4.8	5.4	1.5	10.2	6.80	5.4	3.60
15	6.6	4.3	3.9	10.9	2.79	4.3	1.10
16	7.6	8.0	4.7	15.6	3.32	8.0	1.70
17	5.6	7.8	4.5	13.4	2.98	7.8	1.73
18	6.8	5.1	3.8	11.9	3.13	5.1	1.34
19	6.0	3.1	4.1	9.1	2.22	3.1	0.76
20	5.9	4.0	2.8	9.9	3.54	4.0	1.43
21	5.9	6.1	3.1	12.0	3.87	6.1	1.97
22	6.6	5.9	5.1	12.5	2.45	5.9	1.16
23	7.2	6.1	3.6	13.3	3.69	6.1	1.69

RSD% ≤ 1.5 for all % mass loss values at the steps A, B and C.

The representation obtained using these data evidenced a good separation of all the samples studied into two different main clusters.

On the basis of studies published by G. Szoor [13], the separation of the samples into two classes can probably be attributed to the different age of bones from different burials found in the same archaeological site.

In actual fact, in our opinion, computing the first coefficient as the sum of (A + B) is not fully justified, as the moisture content is an unreliable parameter since, as the samples are conserved in closed but not airtight glass containers at ambient temperature, their moisture content found using TG may reflect the original one only in part.

**Figure 3:**
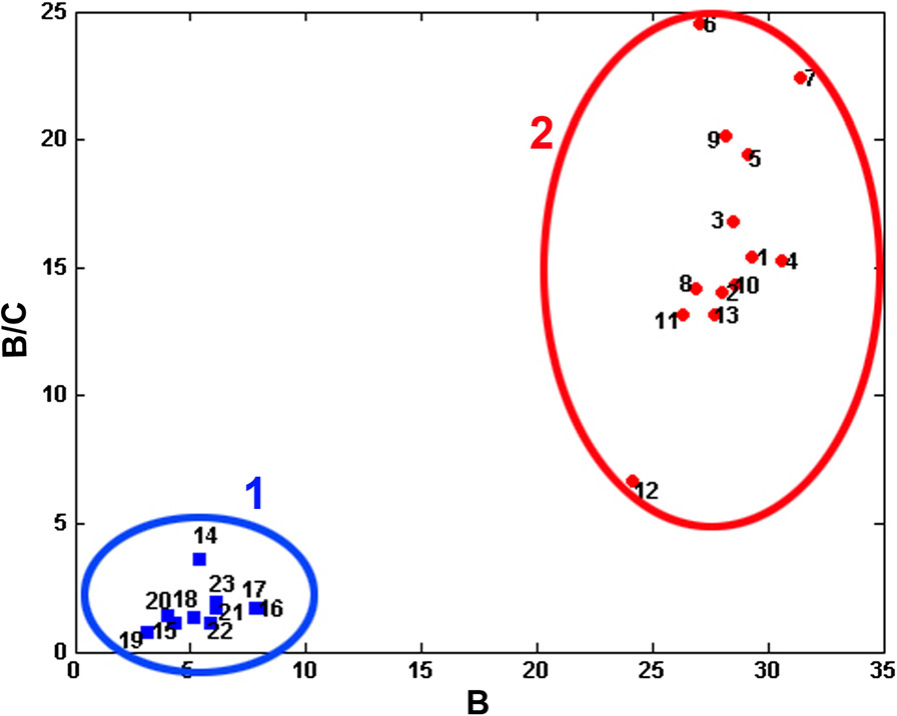
**Graphical representation of thermal data according to the two parameters obtained by simplifying the method of Szoor [**[13]**], considering step B only instead of (A + B).**

It would seem more realistic to use the mass loss of the single step (B) instead (A + B), and of course the step C, that is (see Table 2) to use the simplified alternative parameters (B) and (B/C). In Figure 3 it is indeed apparent that by substituting the value of the single step (B) for the sum of (A + B), a separation of the samples into two clusters is obtained which corresponds in practice to those shown in Figure 2. Analysing the data in Table 2 and their representations in Figures 2 and 3 (in practice, respectively, the representation according Szoor and that with the small modification introduced by us above) and lastly the two clusters that in both cases are quite apparent, it is immediately obvious that the clear-cut separation of the samples into two clusters is due to the fact that, in those samples containing a lower percentage of total collagen the total carbonate percent is instead higher.

According to Szoor, this type of sample, grouped together in cluster 1 in these figures, is older than that grouped in cluster 2 in the same figures. Indeed the latter display a lower carbonate percentage but a higher percentage of collagen.

Moreover, in a well-known article, Bonucci and Graziani [14] focused essentially on the study (also of a thermogravimetric nature) of the inorganic components of different types of bones, both fossil and modern. In particular, these authors observed a reduced weight loss in the TG curves of fossil and ancient bones compared with more recent bones, which may essentially be accounted for by a lower percentage content of organic matter in the former than in the latter.

On the other hand, above approximately 600°C, smaller decomposition steps may be observed, all ascribable to the thermal decomposition of the carbonate materials. The DTG peaks associated with these steps occur at different temperatures, between about 600 and 850°C, depending on the sample. In particular, between 600 and 750°C, one or two carbonate decomposition steps are observed. This kind of carbonate is named “secondary calcium carbonate” [4]. Haas and Banewicz [15] pointed out that the samples containing larger amounts of carbonate (essentially secondary calcium carbonate) and less collagen are those that are more mineralized (and thus older). The same observation was made also by Lozano et al. [16], E. Bonucci and G. Graziani [14] and above all by G. Szoor [13], who in practice, partly based his dating method on this observation.

Lastly, between about 750° and 850°C, a small decomposition step is observed, the large DTG peak of which may be assigned to about 800–850°C. The latter step is nevertheless very important as, according to Haas and Banewicz [15], it must be ascribed to bone-apatite breakdown and thus to the decomposition of the carbonate ion initially present in the apatite lattice and not to the carbonate present in the bone in a separate phase such as the “secondary” calcium carbonate contained in the fossil bone, which is indeed, as explained above, the one in which decomposition occurs in steps at a slightly lower temperature. These authors claim that the secondary calcium carbonate is present in greater quantities in the phases of the more mineralized bones, whereas, the carbonate ion present in the apatite lattice is contained also in sound bone and, according to this author, it is this that could actually be used for dating purposes.

All this is in complete agreement with our results since, as pointed out in Table 1 we thus tried to implement the ideas of Haas and Banewicz [15], namely to plot in a binary diagram the percentage mass loss due to secondary calcium carbonate, which decomposes between 600 and 750°C versus the mass loss observed at 750 and 850°C due to the breakdown of the carbonate originally present in the apatite lattice (see Table 3).

**Table 3:** Proposed parameters based on the thermal data for the two carbonate loss substeps

**Sample number**	**Substep c(1)**	**Substep c(2)**	**c(1)/c(2)**	**c(2)/c(1)**	**c(1) + c(2)**
1	1.5	0.4	3.75	0.267	1.9
2	1.7	0.3	5.67	0.176	2.0
3	1.4	0.3	4.67	0.214	1.7
4	1.6	0.4	4.00	0.250	2.0
5	1.5	0.0	∞	0.000	1.5
6	1.1	0.0	∞	0.000	1.1
7	1.4	0.0	∞	0.000	1.4
8	1.5	0.4	3.75	0.267	1.9
9	1.4	0.0	∞	0.000	1.4
10	1.6	0.4	4.00	0.250	2.0
11	2.0	0.0	∞	0.000	2.0
12	2.6	1.0	2.60	0.385	3.6
13	2.1	0.0	∞	0.000	2.1
14	0.0	1.5	0.00	∞	1.5
15	3.1	0.8	3.87	0.258	3.9
16	3.5	1.2	2.92	0.343	4.7
17	3.5	1.0	3.50	0.286	4.5
18	3.0	0.8	3.75	0.267	3.8
19	3.3	0.8	4.12	0.242	4.1
20	2.8	0.0	∞	0.000	2.8
21	2.7	0.4	6.75	0.148	3.1
22	3.6	1.5	2.40	0.417	5.1
23	2.7	0.9	3.00	0.333	3.6

RSD% ≤ 1.5 for all % mass loss values.

**Figure 4:**
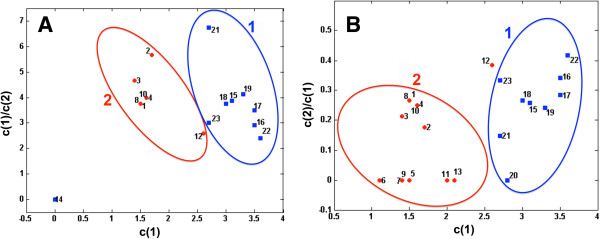
**Graphical representation of the differences in carbonate loss of the analyzed samples reported in Table** 3**as: (A) c(1)/c(2) vs c(1) (indicated in the text as representation [B]); (B) c(2)/c(1) vs c(1) (described in the text as representation [C]).**

**Figure 5:**
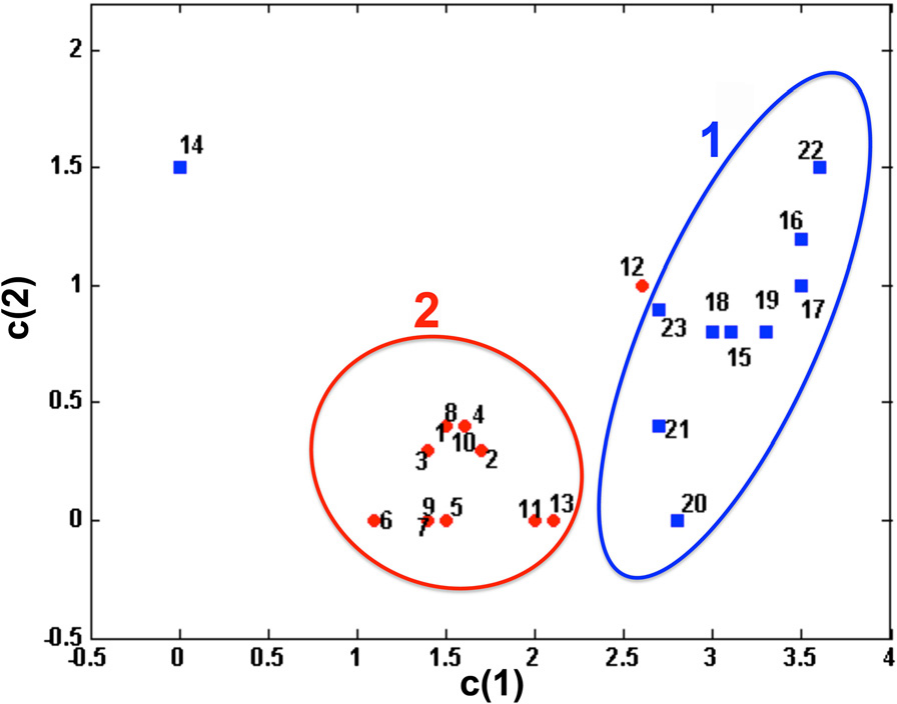
**Graphical representation of the differences in carbonate loss of the analyzed samples, reported in Table** 3**, as c(2) vs c(1), labeled as representation [A], as cited in the text.**

**Figure 6:**
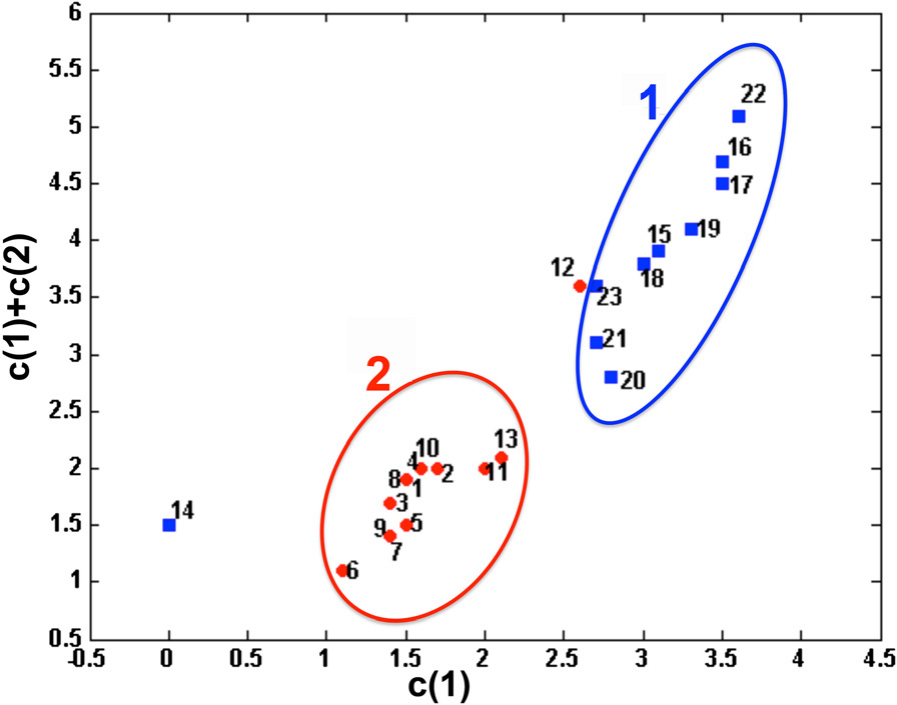
**Graphical representation of the differences in carbonate loss of the analyzed samples, reported in Table** 3**, as: c(1) + c(2) vs c(1), labeled as representation [D], as cited in the text.**

Several different types of possible representation were attempted: [A], c(2) versus c(1), or [B], c(1)/c(2) versus c(1), or [C], c(2)/c(1) versus c(1), and lastly [D], c(1) + c(2) versus c(1). In the cases [B] and [C], since several of the computed values are zero, from the c(1)/c(2), or c(2)/c(1), quotients are therefore obtained with an infinite value (see Table 3); that is, in practice, that cannot be represented in a simple binary diagram, as shown in Figure 4(A) and 4(B). In the cases [A] and [D], on the other hand, all the values referring to the 23 samples considered can be represented graphically (see Figures 5 and 6).

From the graphical standpoint the better of the last two representations is without doubt that in Figure 6, corresponding to the representation [D]. However, the information that may be inferred from this is always the same, whatever the type of representation used. The vast majority of the samples may essentially be grouped into the customary two clusters which respectively identify the more highly mineralized samples, i.e. those which a higher calcium carbonate percentage content (and therefore older) and the less mineralized (and younger) ones with a lower percentage content of secondary calcium carbonate. Nevertheless, unlike what happens in the representation using the parameters proposed by Szoor [13], which involves also the collagen content as well as that of the carbonate, two of the 23 samples examined (number 12 and 14), in the latter representations are distant from the respective cluster to which they should more closely belong if we refer to Szoor’s more correct representation. It may ultimately be concluded that, on the basis of the carbonate content alone, which is subdivided into carbonate originally contained in the hydroxyapatite, which then decomposes at higher temperatures (over about 750°C) and reformed calcium carbonate, which decomposes at lower temperatures (between about 650 and 750°C), as proposed by Haas and Banewicz [15], the distinction between samples of different degrees of mineralization provides a truly correct result in the majority of cases, although not always. Indeed in a small but non negligible number of cases this type of representation could also lead to erroneous results. Also in this case, however, essentially two clusters emerge (Figure 6) which incompletely but all in all agree quite well with those obtained using Szoor’s method (see Figures 2 and 3) and confirm via a different approach the results we obtained applying the method of the latter author. Therefore, in agreement with H. Haas, but also other authors [16,17], it may also be claimed that the samples containing a higher percentage of secondary calcium carbonate are generally the most mineralized and therefore older; if the clusters in Figure 6 are compared with those in Figures 2 or 3, and taking into account the thermogravimetric data contained in Table 1, it is apparent that also the latter (i.e. those belong to cluster 1), are those in which the mass loss due to collagen decomposition is lower.

At this point we decided we had collected sufficient information about which parameters could actually prove useful to attempt a chemometric description of all the fossil bone samples studied making use of the more significant thermogravimetric data emerging from the previous two-dimensional representations and the relations observed between several of the thermogravimentric data and the greater or lesser degree of mineralization of the bone sample. Using the following thermal data: the total collagen decomposition mass loss, the two respective carbonate decomposition mass losses (i.e., that of the secondary calcium carbonate and that of the carbonate originally present in the apatite lattice), and lastly the TG residue at 1000°C, it was possible to assemble a suitable table of numerical data, that could be used as a dataset for the chemometric processing of TG data after auto-scaling (Table 4); 6-fold cross-validation [18] was used to select the optimal complexity of the model, which was found to comprise 2 components.

**Table 4:** **Data matrix used for PCA containing the values of the thermal data reported in Table**1**after autoscaling**

**Sample number**	**Step (b)**	**Substep c(1)**	**Substep c(2)**	**% TG residue at 1000°C**
1	0.950	-0.698	-0.257	-0.907
2	0.837	-0.486	-0.461	-0.890
3	0.880	-0.805	-0.461	-0.764
4	1.062	-0.592	-0.257	-1.086
5	0.932	-0.698	-1.073	-1.006
6	0.751	-1.124	-1.073	-0.782
7	1.131	-0.805	-1.073	-0.907
8	0.742	-0.698	-0.257	-0.791
9	0.854	-0.805	-1.073	-0.863
10	0.889	-0.592	-0.257	-0.952
11	0.690	-0.167	-1.073	-0.666
12	0.500	0.472	0.967	-0.586
13	0.811	-0.060	-1.073	-0.782
14	-1.118	-2.294	1.987	1.443
15	-1.214	1.004	0.559	1.122
16	-0.893	1.429	1.375	0.710
17	-0.911	1.429	0.967	0.907
18	-1.144	0.897	0.559	1.113
19	-1.317	1.217	0.559	1.318
20	-1.240	0.685	-1.073	1.327
21	-1.058	0.578	-0.257	1.086
22	-1.075	1.536	1.987	0.961
23	-1.058	0.578	0.763	0.996

**Figure 7:**
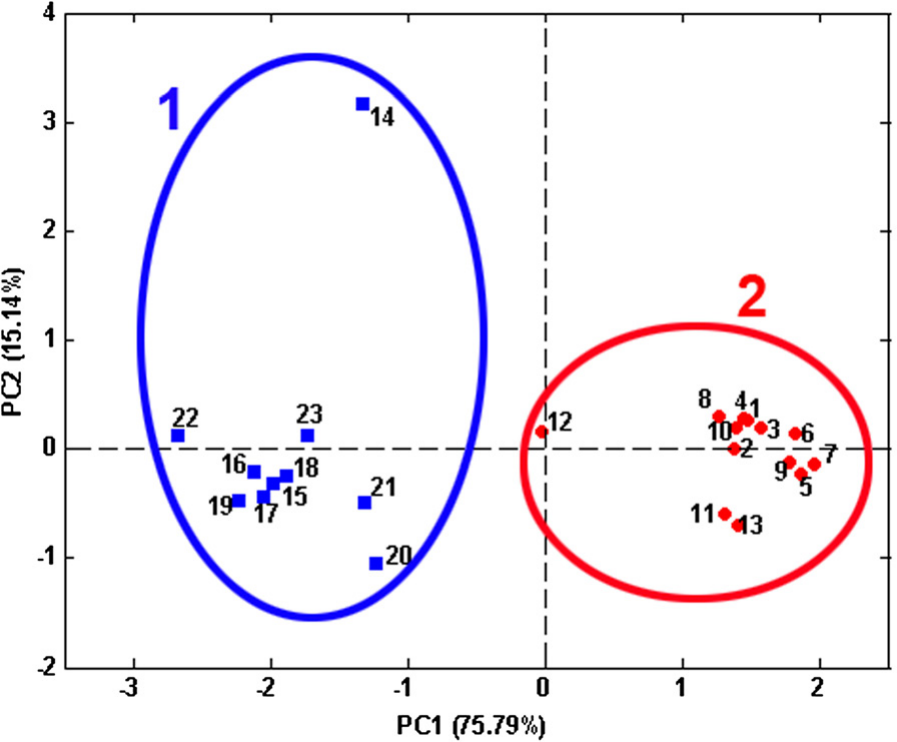
**PCA analysis of the thermal data reported in Table** 4**: scores plot.**

As expected, the representation evidenced (Figure 7) a good separation of all the studied samples into two main clusters, which respectively contain exactly the same bone samples as those identified using two parameters proposed by Szoor and represented in Figures 2 and 3.

**Figure 8:**
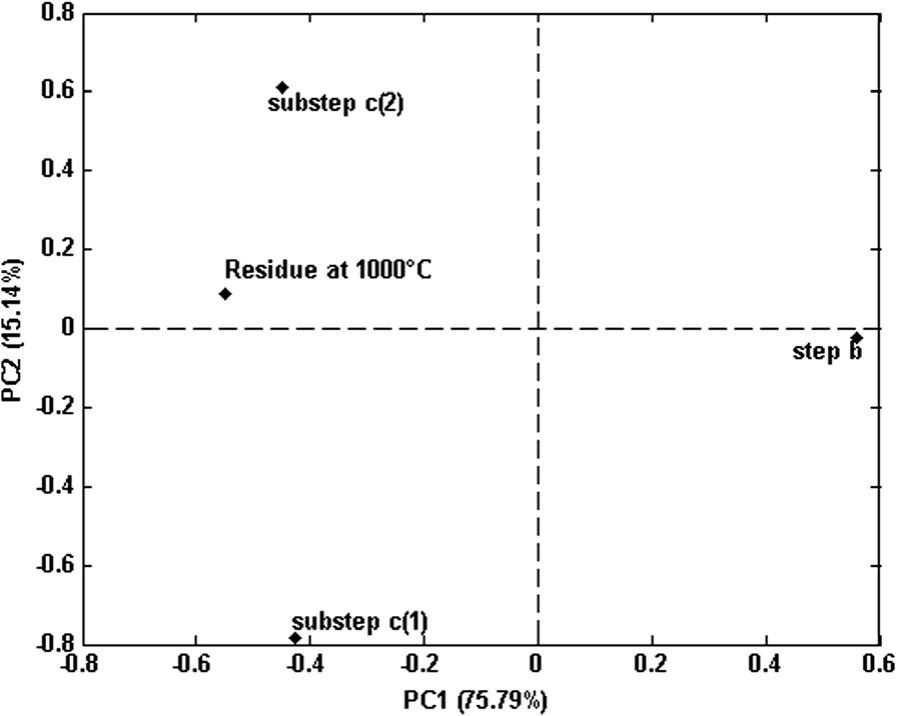
**PCA analysis of the thermal data reported in Table** 4**: loadings plot.**

It thus follows that, also in this type of representation, the fossil bone samples belonging to cluster 1 are the more mineralized and therefore older while those belonging to cluster 2 are less ancient. This observation can be confirmed by inspecting the values of the loadings, reported in Figure 8: indeed, the scores plotted in Figure 7 show how the two clusters are separated along PC1, so that the loadings along this component should account for the observed differences.

In particular, samples in cluster 1, which are found at negative values of the PC, are characterized by higher values of the residue at 1000°C and of the two mass losses at carbonate TG substeps c(1) and c(2). On the other hand, samples from cluster 2 show a greater mass loss for TG step (b).

**Figure 9:**
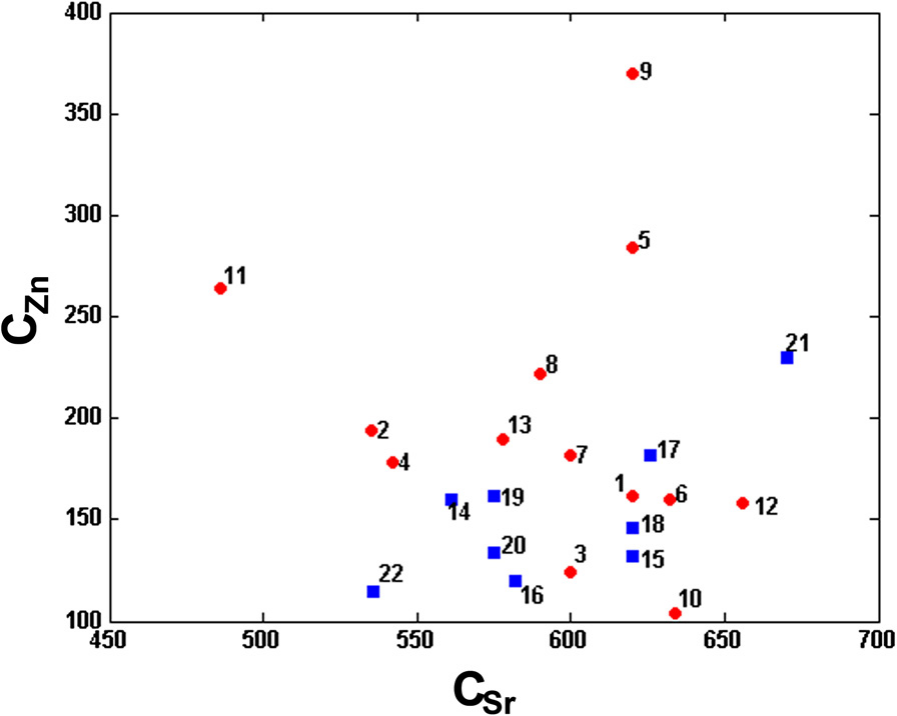
**Graphical representation of ICP data: Sr *vs *** **Zn distribution in bone samples from cluster 1 (blue squares) and 2 (red circles).**

Lastly, since the relative ratio of several elements contained in fossil bones is also of considerable archaeo-paleontological interest [9,10], the Ca, Sr and Zn content of the samples tested was measured by ICP spectroscopy. The results obtained are summarized in Table 5. The relation between the Sr and Zn found content is illustrated graphically in Figure 9.

**Table 5:** Results of ICP analysis: Zn, Ca and Sr content and their ratios

**Sample number**	**Zn (ppm)**	**Ca (ppm)**	**Sr (ppm)**	**Sr/Zn**	**Sr/Ca**
1	162	4.24 10^5^	620	3.83	1.46 10^-3^
2	194	3.80 10^5^	535	2.76	1.41 10^-3^
3	124	4.28 10^5^	600	4.84	1.40 10^-3^
4	178	4.09 10^5^	542	3.04	1.33 10^-3^
5	284	4.15 10^5^	620	2.18	1.49 10^-3^
6	160	4.20 10^5^	632	3.95	1.50 10^-3^
7	182	4.09 10^5^	600	3.30	1.47 10^-3^
8	222	4.32 10^5^	590	2.66	1.37 10^-3^
9	370	4.40 10^5^	620	1.68	1.41 10^-3^
10	104	4.16 10^5^	634	6.10	1.52 10^-3^
11	264	4.22 10^5^	486	1.84	1.15 10^-3^
12	158	4.36 10^5^	656	4.15	1.50 10^-3^
13	190	4.20 10^5^	578	3.04	1.38 10^-3^
14	160	4.02 10^5^	561	3.51	1.40 10^-3^
15	132	4.14 10^5^	620	4.70	1.50 10^-3^
16	120	4.26 10^5^	582	4.85	1.37 10^-3^
17	182	4.20 10^5^	626	3.44	1.49 10^-3^
18	146	4.08 10^5^	620	4.25	1.52 10^-3^
19	162	4.41 10^5^	575	3.55	1.30 10^-3^
20	134	4.30 10^5^	575	4.29	1.34 10^-3^
21	230	4.09 10^5^	670	2.91	1.64 10^-3^
22	114	4.12 10^5^	536	4.70	1.30 10^-3^

RSD% for all determinations is ≤ 1.3.

**Figure 10:**
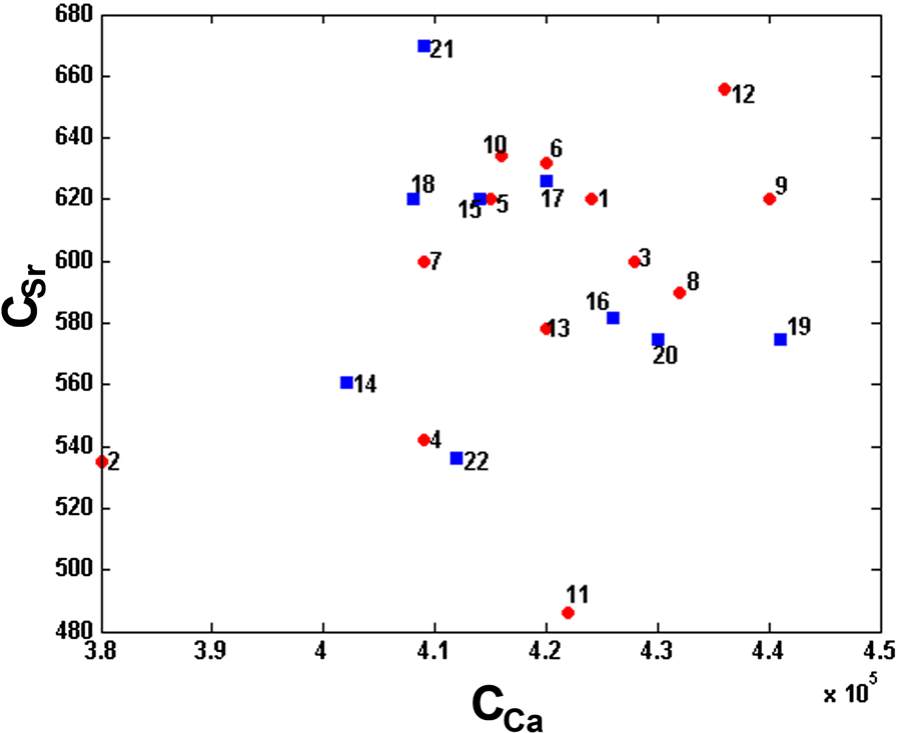
**Graphical representation of ICP data: Sr *vs *** **Ca distribution in bone samples from cluster 1 (blue squares) and 2 (red circles).**

It should be noted that, except in 3–4 cases, the Sr/Zn ratio in the various samples is somewhat similar (see Table 5): the Zn content is rather low, while that of Sr is generally medium-high. This would seem to point to a mainly vegetarian diet with a significant contribution made by mollusks [1]. However, more detailed observation reveals that the 3 – 4 samples in which the zinc content is significantly higher all belong to cluster 2 in Figure 7. On the other hand, although not to any significant extent, nearly all the samples belonging to cluster 2 tend to have a slightly higher Zn content than the cluster 1 samples. Also this observation, therefore, which should be regarded with great caution, seems to indicate that the samples of individuals belonging to cluster 2, that is, those for which the preceding observations tended to indicate as having a lower old age than those belonging to cluster 1 probably belong to individuals that followed a diet which, although essentially vegetarian, was nevertheless slowly beginning to change. The 3 – 4 individuals with a relatively high Zn content actually seem already to be outside this diet and to have adopted a more varied diet richer in mollusks and perhaps even containing meat. Lastly, the Sr *vs* Ca content values are plotted in the graph in Figure 10. In the latter case, the representative points of all the samples lie in relatively homogeneous and random groupings.

On the basis of literature reports [1], a possible interpretation could however be that the society to which these individuals belongs was still not significantly differentiated into distinct social classes.

### Experimental

Several samples of human fossil bones from different parts of human body, after being gently brushed and coarsely ground, were subjected to TG and DTG analysis [19] under an air stream (100 cm^3^ min^-1^) with a heating rate of 10°C min^-1^ using a Mettler 50 thermobalance, coupled with a Mettler TG 10-TA processor system (Mettler Toledo Inc., Hightstown, NJ). About 10 mg of each sample were gently flaked, placed in an alumina crucible, weighed and then analyzed without further pretreatments. Main TG-DTG data were processed by PCA analysis using in-house routines written in Matlab (The Mathworks Inc., Natick, MA).

Plasma emission (ICP) measurements were carried out using a Varian Liberty 150 plasma spectrophotometer (Varian Inc., Palo Alto, CA), performing the mineralization of sample using a MDS-61 D microwave mineralizer (CEM Corporation, Buckingham, UK) and treating the samples with nitric acid and hydrogen peroxide (1:1 V/V) [20]. In particular, microwave-assisted extraction was carried out working at a power of 70 W for 15 mins, as suggested in the literature [21,22].

## Conclusions

In this study, the potential of coupling TG, ICP and chemometrics to investigate archaeological findings has been demonstrated. In this framework, it must be stressed that the scope of the present research was not to provide an exact dating of the analyzed samples nor to build any archaeometric scale as it was still not possible to make a comparison with results obtained by subjecting the same samples to an already validated dating method. However, the experimental data obtained using thermogravimetry (TG-DTG), processed by chemometrics, allows us to differentiate all the fossil bone samples analyzed into two separate clusters, that were not so different from those obtained using the parameters proposed by G. Szoor [13], or Haas and Banewicz [15], but in any case better defined. Therefore, on the basis of the criteria set by the above-cited researchers, with which also other authors substantially agree [14,16], the two identified clusters of samples should be characterized by different degrees of antiquity. In the present research this idea was confirmed not only by the differences in the (% collagen /% carbonate) ratio, but to a large extent (although not completely) also by the observation regarding the carbonate thermal decomposition, i.e. by the difference in the (% secondary calcium carbonate +% calcium carbonate originally contained in the apatite lattice)/(% secondary calcium carbonate) ratio. Lastly, also the evaluation of the ratios of the Sr and Zn quantities, obtained by ICP coupled plasma emission, also seem to confirm this conclusion even though, in the latter case, in a largely qualitative fashion.

## Abbreviations

PCA: Principal component analysis; TG: Thermogravimetry; DTG: Differential thermogravimetry; ICP: Inductively coupled plasma emission spectroscopy.

## Competing interests

The authors declare that they have no competing interest.

## Authors’ contributions

MT designed the study and performed the TG analysis, FM was in charge of the chemometric data processing, AC provided the palaeontological interpretation, while LC conducted the bibliographic search and supervised the work. All authors read and Sd the final manuscript.
